# Physical activity affects DNA methylation-derived inflammation markers in a community-based Parkinson's disease study

**DOI:** 10.1016/j.bbih.2025.101014

**Published:** 2025-05-22

**Authors:** Yang Cheng Hu, Keren Zhang, Kimberly C. Paul, Jin Zhou, Jeff M. Bronstein, Cynthia D.J. Kusters, Beate R. Ritz

**Affiliations:** a Department of Epidemiology, UCLA Fielding School of Public Health, Los Angeles, CA, USA; b Department of Neurology, UCLA David Geffen School of Medicine, Los Angeles, CA, USA; c Cousins Center for Psychoneuroimmunology, University of California, Los Angeles, Los Angeles, CA, USA; d Jane and Terry Semel Institute for Neuroscience and Human Behavior, Department of Psychiatry & Biobehavioral Sciences, University of California, Los Angeles, Los Angeles, CA, USA; e Department of Environmental Health Sciences, UCLA Fielding School of Public Health, Los Angeles, CA, USA

**Keywords:** Parkinson's disease, Physical activity, Exercise, DNA methylation proxies, Chronic inflammation

## Abstract

**Introduction:**

Past studies have connected physical activity (PA) and Parkinson's disease (PD) to chronic inflammation. We use DNA methylation-derived (DNAm) proxies for inflammation to investigate the relationship between PA and chronic inflammation among PD patients.

**Methods:**

We collected demographics, lifestyle, and PA status information by interviewing 555 PD patients enrolled in the Parkinson's Environment and Gene (PEG) studies. We used the epigenetic clock website to generate DNAm proxies and performed principal component analysis (PCA) of 22 DNAm cytokine proxies. Using the Mann-Whitney *U* test, we compared the PC scores of active and sedentary patients. For PCs associated with PA status, we examine associations between PA status, the amount of PA, and PCs for DNAm cytokines using permutation-based tests.

**Results:**

Compared to sedentary PD patients, those who reported strenuous PA have lower levels of c-reactive protein (CRP; p < 0.01) and higher levels of Fc receptor-like 2 (FcRL2; p = 0.02). Patients who engaged in moderate PA have lower levels of C-X-C motif chemokine ligand 9 (CXCL9; p = 0.03), 10 (CXCL10; p = 0.02), and 11 (CXCL11; p = 0.01). Among active patients, strenuous PA is non-linearly (convex-shape) associated with the proportions of natural killer cells (NK; p = 0.02) and CD8T cells (p = 0.05) and negatively associated with CRP levels (p = 0.02). We also observe a non-linear association between moderate PA and monocyte counts (p = 0.02).

**Conclusion:**

PA may benefit PD patients by reducing chronic inflammation. We also found that strenuous PA may increase the proportions of NK and CD8T cells, though further effort is needed to confirm potential shifts in immune cell subtypes.

## 1 Introduction

Parkinson's disease (PD) is a chronic disease with motor and non-motor dysfunction that progresses in highly heterogeneous ways (Greenland et al., 2019) and likely involves complex pathological pathways, including chronic inflammation. More and more evidence has been accumulating from in-vitro (Le et al., 2001; Gao et al., 2002) and animal models (Cebrián et al., 2014; Leal et al., 2013), human PET-scan imaging (Gu et al., 2017; Marsland et al., 2015), and post-mortem PD brains (Hartmann, 2004; Hald and Lotharius, 2005) that chronic inflammatory responses and microglia activation are part of the pathology. Over the past two decades, systemic inflammation and changes in immune cell populations in peripheral tissues have also been linked to PD risk and may play a critical part in PD progression (Williams et al., 2022; Contaldi et al., 2022). For example, pro-inflammatory T cells (e.g., Th-1 and Th-17) can infiltrate across the blood-brain barrier (BBB) and release pro-inflammatory cytokines such as interferon-gamma (IFN-γ), tumor necrosis factor-alpha (TNF-α), and certain members of the interleukin (IL) family (e.g., IL-17, IL-21, and IL-22) that stimulate microglia to initiate neuroinflammation (Wang et al., 2015; McGeer and McGeer, 2004).

Lifestyle and behavior are potential upstream factors propagating PD risk (Poewe et al., 2017; Shih et al., 2018; Paul et al., 2019; Hernán et al., 2002). Among these, moderate-to-vigorous levels of physical activity (PA) are amongst the strongest modifiable protective factors against the onset and progression of PD (Paul et al., 2019; Thacker et al., 2008; Shih et al., 2016; Yang et al., 2015; Xu et al., 2010; Tsukita et al., 2022; Amara et al., 2019). Both animal and human studies have shown that long-term PA is linked to an increased anti-inflammatory capacity in the general population (Frankel et al., 2012; Khalili et al., 2013; Dorans et al., 2016; Souza et al., 2017; Luo et al., 2024; Sieczkowska et al., 2021). However, there is little evidence for long-term PA modulating PD through chronic inflammation. Intervention studies suggest that after a 2-to-3-months moderate-intensity balance (Szymura et al., 2020) or cycling training (Zoladz and Majerczak, 2014), PD patients' serum TNF-α levels are lower than at baseline. These studies also reported increased brain-derived neurotrophic factor (BDNF) in serum compared to baseline (Szymura et al., 2020; Zoladz and Majerczak, 2014). Nonetheless, more evidence is needed to establish whether longer-term PA as part of daily life routines can affect inflammation among PD patients, as clinical trials mainly have focused on short-to-mid-term exercise programs (4 weeks–2 months) specifically designed for highly motivated patients rather than reflect activities that patients can more easily integrate into their daily routines, as exercise studies tend to enroll participants very selectively.

To address this, we use DNA methylation-derived (DNAm) proxies to estimate the proportions of six immune cell types and the abundance of over 20 inflammatory cytokines to investigate the relationship between overall daily PA and chronic inflammation in PD. These proxies have previously been shown to be reliable markers for inflammation, as they allow for high-validity immune cell quantification (Houseman et al., 2012; Baron et al., 2018) and reflect proteomic alterations associated with chronic diseases (Houseman et al., 2012; Gadd et al., 2022; Horvath, 2013a). Moreover, polygenic DNAm scores for cytokines have shown to have stable longitudinal trajectories and even to outperform repeated protein measurements in serum (Stevenson et al., 2021; Conole et al.).

Here, we investigate the relationship between self-reported long-term PA and peripheral immune profiles using DNAm proxies among PD patients recruited in a community-based study. We hypothesize that patients who engage in any amount of PA, compared to those who are mostly sedentary, exhibit a different distribution in estimated immune cell proportions and cytokine levels and that the observed patterns reflect an overall reduction in chronic inflammation.

## 2 Method

### 2.1 Study population

We analyzed data collected from participants in the Parkinson's Environment and Gene (PEG) study (details have been described elsewhere (Kang et al., 2005; Duarte Folle et al., 2019; Wang et al., 2014; Ritz et al., 2016; Paul et al., 2016)). Briefly, the PEG study is an ongoing research project investigating the connections between genetics, lifestyle, and environment and the development and progression of Parkinson's disease (PD). Over the past two decades, the study has gathered information on medical histories, repeatedly conducted neurological examinations and cognitive assessments, and collected biosamples.

The PEG study first collected information on community-based PD patients and a control group from the general population of these same communities. Later, it expanded to track the progression of PD in two waves, PEG1 and PEG2. The study population consists of individuals living in Fresno, Tulare, and Kern counties in central California. In PEG1, newly diagnosed PD cases were recruited within three years of diagnosis, whereas in PEG2, this time was extended to five years. Additionally, eligible cases had to reside within the study area, have a baseline health status allowing them to live independently, visit a temporary study clinic for the exams, and have received a clinical diagnosis of possible, probable, or definite PD from a UCLA study movement disorder specialists, and provided informed consent. A total of 357 participants in PEG1 and 470 in PEG2 were eligible and enrolled after the baseline exams.

### 2.2 Data collection

#### 2.2.1 Lifestyle interview and information

A wide range of information was gathered from participants during interviews and physical exams, including (but not limited to) demographic characteristics, physical activity (PA), other lifestyle factors, and PD progression data. Demographic information collected included age (at the time of the interview), sex, smoking status (past, current, or non-smokers), and age at PD diagnosis.

Participants were asked to report the average number of days per week and the average number of hours per day during which they engaged in mild, moderate, or vigorous physical activity. Types of PA reported could be related to leisure (e.g., jogging, swimming) or work (e.g., crop harvesting, gardening), and definitions of activity intensity were provided during the interview. The definition of the “current” period differed between PEG1 and 2. In PEG1, participants were asked to recall their average physical activity during senior years (≥ 65 y/o) or the middle-age period (i.e., 45 to 65 y/o) for those younger than 65 y/o. In contrast, the current PA in PEG2 asked participants about their PA in the past 12 months.

#### 2.2.2 Motor function examination among PD patients

Repeated neurological examinations of PD patients were conducted by our UCLA study movement disorder specialists, who employed the Unified Parkinson's Disease Rating Scale (UPDRS) and later the new version sponsored by the Movement Disorder Society (MDS-UPDRS). We used corrected scores for comparability to the original UPDRS assessments (Goetz et al., 2008). Most participants in the PEG study (86.5 %) were examined during an overnight “wearing-off” period after their last PD medication. Time since the last levodopa dose was also recorded for PD patients assessed who were in an “on” period (i.e., did not refrain from using medication the day of the exam).

#### 2.2.3 DNA methylation profiling and DNAm proxies

The PEG study collected peripheral whole blood samples for DNA extraction. At the time of conducting methylation arrays with the Illumina Infinium HumanMethylation450 BeadChip array (Illumina 450K array), 555 PD patient samples had been collected and had passed quality assurance (n = 332 from PEG1; n = 223 from PEG2). To ensure the quality of signal readings, we excluded samples with a mean detection P value > 0.01 and imputed missing values with the k-nearest neighbor (KNN) algorithm. We also used beta-mixture quantile normalization (BMIQ) (Teschendorff et al., 2013) to stabilize methylation beta values. Then, we filtered out cross-reactive probes, leaving us with a final CpG count of 352,325 for proxy marker derivation. We used the epigenetic clock website (clockfoundation.org) (Lu et al., 2022; Horvath, 2013b) to generate immune cell proportions for granulocytes, monocytes, B cells, total CD4T, total CD8T, and natural killer cells with the Houseman methods (Houseman et al., 2012). The website algorithm also generated 47 DNAm proxies for molecules involved in chronic inflammation, neuron development and damage response, insulin sensitivity, antibody production, or other pathways. After reviewing the biological function of each marker, six immune cell types and 27 inflammation markers were selected for analysis (see Table S-1 for the full list of inflammation markers).

### 2.3 Metabolic equivalent score estimation

The method for deriving MET scores is the same as described in our previous reports (Shih et al. (2016), Paul et al. (2019), and Hu et al. (2024)). Vigorous-level activities were assigned an 8 MET (per hour) and moderate-level a 4 MET (per hour) score. The mild PA category was set to 0, as previous studies suggested mild PA does not contribute to PD prevention (Thacker et al., 2008; Yang et al., 2015). We then converted MET into MET-hour per week (MET-hr/wk) by multiplying the reported frequencies of each activity. For example, if a participant reported that they spent 2 h every day on vigorous activity each week, the MET-hr/wk value would be calculated as 8(MET) × 2(hours) × 7(times per week) = 112 MET-hr/wk. Lastly, we winsorized the MET-hr/wk at the top 1% to account for records with improbable MET values. To further increase the exposure contrast, we also calculated strenuous PA as MET-hr/wk separately (i.e., we only included vigorous-level activities and set the weight for all other activities to zero to calculate the MET score).

### 2.4 Statistical analysis

We generated residuals by fitting a series of random intercept models for each methylation measure (both inflammation proxies and immune cell proportions) based on covariates and using PEG waves (PEG1 or 2) as the clustering variable. Covariates included age (in years), sex (male or female), and the baseline UPDRS-III score.

Next, we used principal component analysis (PCA) to reduce the dimensionality of our residual data. We applied both the global Kaiser-Meyer-Olkin (KMO) criterion (Pearson correlation threshold: 0.5) and Bartlett's test of sphericity (p-value threshold: 0.05) prior to PCA to assess whether the residual data is suitable for factor analysis. The KMO criterion was also applied to each cytokine proxy separately, and proxies with a score of less than 0.5 were removed to ensure the stability of the resulting PCs (Figure S-1). We performed PCA after centering and scaling the data, then used the PCAtest package in R to evaluate the overall statistical significance of the PCA and the contributions of each loading variable within a PC using permutation-based tests (p-value threshold for φ and ψ statistics is 0.05) (Vieira, 2012; Camargo, 2022). Finally, we bootstrapped 95% confidence intervals for the amount of variance explained.

We categorized patients into two groups based on their self-reported PA status: zero MET (sedentary) and non-zero MET (active). We then employed Mann-Whitney U tests to examine the association between residuals of estimated immune cell measures, PC scores, and PA status (non-zero vs. zero MET). For DNAm cytokine PCs statistically significantly associated with PA status, we employed another set of Mann-Whitney U tests between the cytokine proxies that significantly contributed to the PCs and PA status to check whether the proxies were individually associated with PA status. We also calculated the difference in median levels of DNAm proxy between the active and the sedentary group (d) to examine the direction of the association.

For active patients only, we fitted quadratic regression models to examine associations between residuals of estimated immune cell measures, PC scores, and MET scores. We used a quadratic term in each model because quadratic regression provided a better fit based on both the Akaike information criterion and patterns of residuals.

We calculated all p-values and 95% confidence intervals for PC performance and random intercept models under a type-I error of 0.05. We completed all data cleaning and analyses with R© version 4.1.1.

## 3 Results

### 3.1 Demographic characteristics

The PEG patient population we included contains 555 participants, for whom we show demographics in Table 1. In both PEG1 and 2, the proportion of men is higher (57.2% in PEG1 and 69.5 % in PEG2). Most of the participants are of European ancestry (76.6%). The distributions/frequencies of age, education, family history of PD, and smoking status are similar in PEG1 and 2. While the average duration of the disease at baseline is shorter in PEG1 (2.09 ± 1.54 years) than in PEG2 (3.82 ± 2.79 years), the distributions of UPDRS-III scores at baseline are similar (21.3 ± 10.9 in PEG1 and 24.1 ± 11.9 in PEG2).

**Table 1:** Demographic characteristics for the PEG study population (n = 555).

	PEG 1 (N = 332)	PEG 2 (N = 223)	All PEG cases (N = 555)
**Gender**			
Male	190 (57.2 %)	155 (69.5 %)	345 (62.2 %)
Female	142 (42.8 %)	68 (30.5 %)	210 (37.8 %)
**Age (years)**			
Mean (SD)	70.5 (10.2)	70.8 (9.2)	70.6 (9.8)
Median [Min, Max]	72.0 [37.0, 90.0]	71.0 [46.0, 92.0]	72.0 [37.0, 92.0]
**European ancestry**			
European	268 (80.7 %)	157 (70.4 %)	425 (76.6 %)
Others	64 (19.3 %)	66 (29.6 %)	130 (23.4 %)
**Family history of PD**			
Yes	50 (15.1 %)	34 (15.2 %)	84 (15.1 %)
No	282 (84.9 %)	183 (82.1 %)	465 (83.8 %)
**Smoking status**			
Non-smokers	177 (53.3 %)	122 (54.7 %)	299 (53.9 %)
Former	140 (42.2 %)	94 (42.2 %)	234 (42.2 %)
Current	15 (4.5 %)	7 (3.1 %)	22 (4.0 %)
**PD duration at interview (years)**			
Mean (SD)	2.1 (1.5)	3.8 (2.8)	2.8 (2.3)
Median [Min, Max]	2.0 [0.0, 9.0]	3.0 [0.0, 15.0]	2.0 [0.0, 15.0]
**UPDRS-III score (points)**			
Mean (SD)	21.3 (10.9)	24.1 (11.9)	22.4 (11.4)
Median [Min, Max]	20.0 [3.3, 68.0]	22.3 [4.0, 71.0]	20.3 [3.3, 71.0]

### 3.2 Sampling adequacy for factor analysis

Overall, the Pearson correlation for the global KMO criterion is 0.78 for the residual data of the 27 inflammatory proxies, indicating suitability for factor analysis. This is also supported by Bartlett's test of sphericity (*χ*^2^ = 18332.79 with df = 351; p < 0.001) and the permutation-based test (ψ = 0.36, p < 0.001; φ = 59.64, p < 0.001), indicating non-random correlations in our residual data. However, the individual-proxy KMO suggests sampling inadequacies in Matrilin 3 (MATN-3; KMO = 0.447), Interleukin 18 receptor 1 (IL18R1; KMO = 0.318), Iron regulated transporter 2 (IRT2; KMO = 0.437), neprilysin (NEP; KMO = 0.402), and Sphingomyelin phosphodiesterase 1 (SMPD1; KMO = 0.371). Therefore, we removed these DNAm proxies before conducting the PCA to ensure the stability of PCs.

**Fig. 1:**
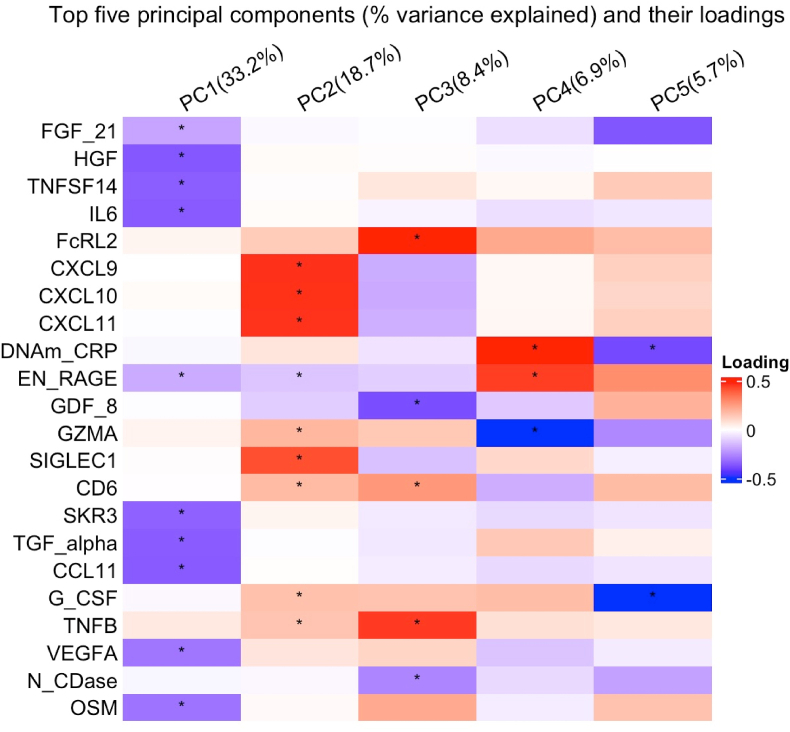
A summary from principal component analysis among 555 PD patients using 22 DNA methylation cytokine proxies: In total, 22 DNA methylation (DNAm) proxies were used for the principal component analysis (PCA). Bootstrapped 95 % CIs suggest that the top five PCs have an eigenvalue of >1 (shown in the column titles). All loadings that significantly contribute to each PC are marked with the star (∗) symbol. (For interpretation of the references to colour in this figure legend, the reader is referred to the Web version of this article.)

The top 7 PCs account for more than 81% of the total variance in the residual DNAm proxy dataset. Bootstrapped 95% CIs of each PC suggest that the top 5 PCs have an eigenvalue of >1 and explain 72.9% of the total variation in the whole residual data (Fig. 1; Table S-2). These PCs account for 33.2% (95 % CI = [31.6%, 34.5%]), 18.7% (95% CI = [17.7%, 20.1%]), 8.4% (95% CI = [7.2%, 9.9%]), 6.9% (95% CI = [6.3%, 7.8%]), and 5.7% (95% CI = [5.2%, 6.3%]) of the variance, respectively. Therefore, only the top five PCs were included in the subsequent analyses.

Permutation tests for the top 5 PCs identified the following DNAm cytokine proxies with the strongest (and statistically significant) loadings in PC 1: FGF 21, HGF, TNFSF14, IL-6, EN-RAGE, SKR 3, TGF-α, CCL-11, VEGFA, and OSM. PC 1 is also the only axis with anti-inflammatory cytokine contributions. Statistically significant loadings on PC 2 were observed for CXCL 9, CXCL 10, CXCL 11, EN-RAGE, GzmA, SIGLEC-1, CD6, G-CSF, and TNF-β. The vector projection from the biplot (data not shown) confirmed that PC 1 and 2 scores covered mostly different groups of inflammatory DNAm-based proxies. For PC 3, the principal contributions to the loadings are from FcRL2, EN-RAGE, GDF-8, CD6, G-CSF, and TNF-β; for PC4 on CRP, EN-RAGE and GzmA, and for PC5 from CRP and G-CSF.

### 3.3 Association between immune cell composition and physical activity

The Mann-Whitney *U* test showed no evidence of a difference in immune cell composition between active and sedentary PD patients (Figure S-2). However, among patients engaged in any amount of strenuous PA, we observed convex trends between the MET score and the residual of CD8T (p = 0.05; Fig. 3-A) and NK (p = 0.02). The same trend was also found for the moderate MET score and monocytes (p = 0.02; Fig. 3-B).

### 3.4 Association between inflammatory markers and physical activity

**Fig. 2:**
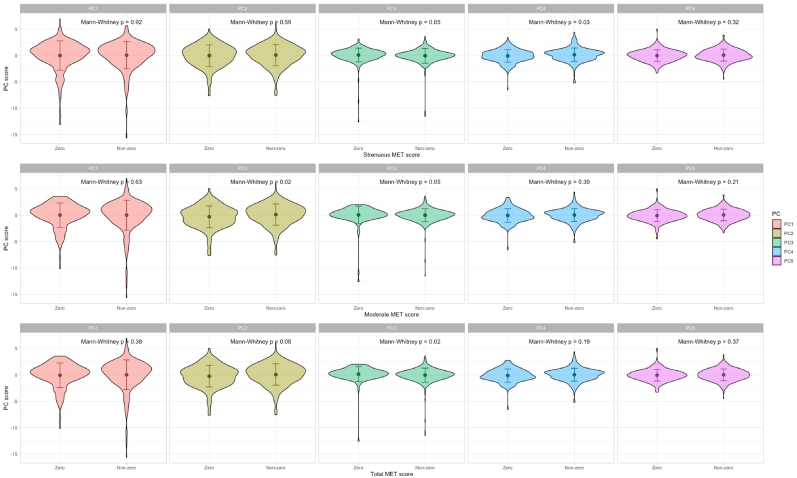
Comparing the top five principal components of the 22 DNAm proxies between the active and the sedentary patient groups: We used Mann-Whitney U tests to determine whether the PC spreads are different between patients who engaged in physical activity (strenuous, moderate, or total) and sedentary patients (i.e., whose estimated MET score is zero). We multiplied PC2, PC3, and PC4 scores by −1 for visual clarity.

We observed differences in PC 3 (p = 0.05; Fig. 2) and PC 4 (p = 0.03) between patients engaged in strenuous PA and those not engaged. Additionally, patients who engaged in moderate PA had different PC 2 (p = 0.02) and PC 3 (p = 0.05) distributions compared to the sedentary group. The distribution of PC 3 also differs by total PA status (p = 0.02).

**Fig. 3:**
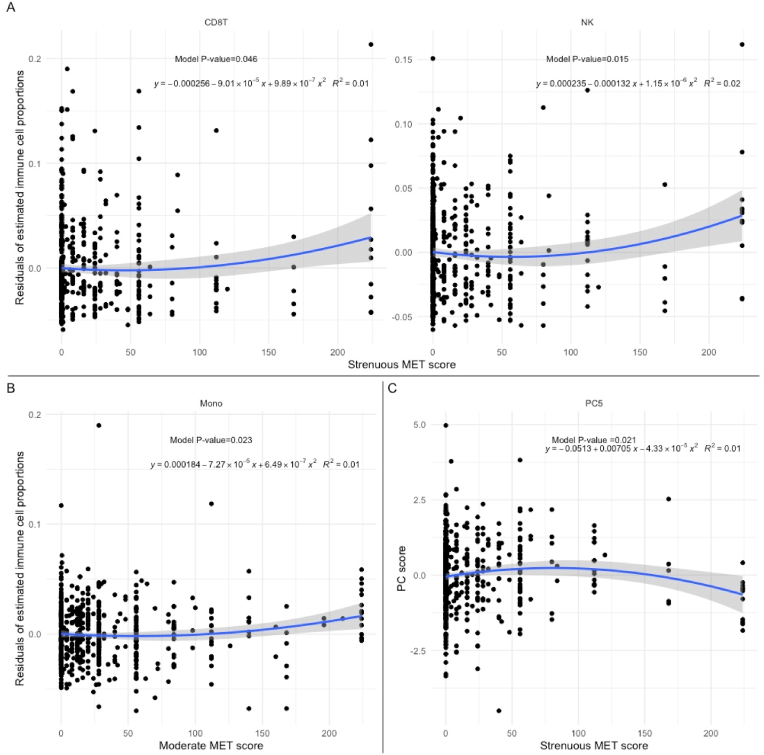
Quadratic associations between metabolic equivalent scores, immune cell types, and principal component scores among active patients group: This graph shows the quantitative relationship between **(A)** estimated proportions of CD8T, natural killer cells, and strenuous MET scores; **(B)** estimated proportion of monocyte and moderate MET scores; **(C)** PC5 and strenuous MET scores. For **(A)** and **(B)**, the y-axis represents the residuals of estimated immune cell proportions after adjusting for age (in years), sex (male or female), the baseline UPDRS-III score, and PEG waves as the clustering variable.

We further tested whether factors with major loadings in these PCs differed by PA status (Table 2). We found that levels of CXCL9 (d = −4.57 × 10^−3^; p = 0.03), CXCL10 (d = −5.01 × 10^−3^; p = 0.02), and CXCL11 (d = −5.19 × 10^−3^; p = 0.01) were statistically significantly lower in patients who engaged in moderate PA, compared to the sedentary group, while engaging in strenuous PA was associated a higher level of FcRL2 (d = 2.08 × 10^−5^; p = 0.02) and lower levels of CRP (−1.09 × 10^−1;^ p < 0.01).

**Table 2:** Associations between individual loadings from significant principal components and physical activity status.

Residuals of Estimated Levels^a^	Stenuous PA		Moderate PA		Total PA	
	Median difference (d)^b^	p-value^c^	Median difference (d)	p-value	Median difference (d)	p-value
**Significant loadings in PC 2**						
CXCL 9			−4.57E-03	0.028099		
CXCL 10			−5.01E-03	0.022911		
CXCL 11			−5.19E-03	0.014226		
EN-RAGE			1.02E-04	0.829152		
GzmA			−1.42E-04	0.52517		
SIGLEC-1			−1.06E-03	0.07107		
CD6			6.95E-05	0.485022		
G-CSF			−1.16E-03	0.4603		
**Significant loadings in PC 3**						
FcRL2	2.08E-05	0.022951	−6.04E-06	0.806827	1.20E-06	0.88819
GzmA	7.23E-05	0.756052	−1.42E-04	0.52517	8.39E-05	0.759491
EN-RAGE	−7.60E-05	0.579952	−1.64E-04	0.829152	−1.80E-04	0.952887
TNF-β	−6.96E-05	0.246963	4.39E-05	0.677179	4.39E-05	0.842475
nCDase	2.07E-04	0.814984	−4.34E-05	0.310154	−1.81E-05	0.37618
**Significant loadings in PC 4**						
CRP	−1.09E-01	0.004237				
EN-RAGE	−1.76E-04	0.579952				
GzmA	4.75E-05	0.756052				

a Residuals of individual markers from linear mixed models.

b Difference in median values between the active and the sedentary group.

c P-value from Mann-Whitney *U* test.

Lastly, we tested whether the PC scores are correlated with numeric MET scores among those who engaged in any PA. We found that PC 5 is non-linearly (or concavely) associated with the strenuous MET-h/wk score (p = 0.02; Fig. 3-C). The corresponding scatter plot suggests that the mean PC 5 score decrease is most pronounced for higher MET-h/wk. Statistically significant loadings suggested a negative association between the CRP residuals and the strenuous MET-h/wk score (ρ = −0.11, p < 0.01; Table S-3).

## 4 Discussions

Our population-based study suggests that being active (versus sedentary) in senior years affects PD patients' longer-term immune and inflammatory profiles. In addition, we observed non-linear relationships between MET scores, the proportion of CD8T and NK, and CRP levels. Our study suggests a potentially beneficial relationship between long-term strenuous and moderate PA and immune responses in PD patients.

One of the most prominent findings in our study is the negative association between strenuous PA and DNAm-derived CRP levels that likely reflects its chronic downregulation. Not only was the DNAm CRP level among patients who engaged in strenuous PA lower than in the sedentary group, but we also observed a quadratic and negative association between CRP and strenuous MET scores. Serum CRP has long been known to act as an indicator of both acute and chronic inflammation and is highly dependent on age (Du Clos and Mold, 2004; Sproston and Ashworth, 2018; Nehring et al., 2023). Past observational (Taaffe et al., 2000; Abramson and Vaccarino, 2002; Ford, 2002; Beavers et al., 2010) and intervention studies (Falck et al., 2019; Kim and Yeun, 2022), as well as meta-analyses (Kim and Yeun, 2022; Rose et al., 2021) consistently reported lower serum CRP among those who regularly engage in vigorous or planned exercise, regardless of body weight, age, or gender. Further, the inverse association between exercise and CRP levels has been reported as being stronger for higher-intensity exercise interventions lasting more than eight weeks in duration (Rose et al., 2021). In terms of mechanism, it has been hypothesized that long-term PA directly inhibits CRP production by reducing upstream pro-inflammatory cytokines (e.g., IL-6, TNF-α) in fat, muscle, and blood mononuclear cells (Kasapis and Thompson, 2005; Metsios et al., 2020). On the other hand, the reduction of CRP could also be an indirect reflection of enhanced antioxidant enzymes (Esposito et al., 2003; Kondo et al., 2005), insulin sensitivity (Mayer-Davis et al., 1998; Gelaye et al., 2010; Kelly et al., 2004), and endothelial function (Kelly et al., 2004).

Considering the individual loadings on PC2 and PA, we found that patients who engaged in moderate PA had lower levels of CXCL9, CXCL10, and CXCL11, known pro-inflammatory chemokines involved in immune cell migration (Callahan et al., 2021; Elemam et al., 2022) and tumor progression (Chang et al., 2013; Liu et al., 2011; Wang et al., 2022). These chemokines are interferon-γ (IFN-γ) inducible ligands that share a common receptor, C-X-C motif chemokine receptor 3 (CXCR3), which mainly triggers NK and CD8T cell mobilization and promotes subsequent inflammatory reactions (Wang et al., 2022; Griffith et al., 2014). Our findings indicate a possible anti-inflammatory effect of moderate PA, which aligns with existing epidemiologic evidence that a regular, moderate exercise habit can strengthen the immune system's response to viral infection by regulating inflammation intensity (Romeo et al., 2010; da Silveira et al., 2021).

CXCL10, also known as interferon gamma-induced protein 10 (IP-10), has recently been suggested as an exercise-reducible myokine due to its reduction after skeletal muscle contraction (Li et al., 2021; Ishiuchi-Sato and Nedachi, 2021; Ishiuchi et al., 2018). These results were further supported by another study that examined the effect of physical activity interventions on inflammatory markers and reported a reduction in CXCL10, along with IL-6 and CXCL1, post-intervention in both elderly humans and mice (three months for the human cohort and ten weeks for the mice) (Marcos-Pérez et al., 2024). This suggests that CXCL10 might be a novel indicator for exercise-induced reduction of inflammation.

On the other hand, we did not see the same association between CXCL 9, CXCL 10, CXCL 11, and strenuous PA status. The effect of strenuous or high-intensity PA on viral infection prevention is more controversial. Past studies reported athletes who often engage in exhaustive training have an increased risk of upper respiratory tract infections (Ruuskanen et al., 2022). However, the underlying mechanism for these observations remains largely unclear.

In terms of PC3 loadings, we found that DNAm FcRL2 levels are higher among patients engaged in strenuous PA than the sedentary patient group, which is also supported by a recent targeted proteomic study (Olvera-Rojas et al., 2023). Among other findings, FcRL2 was reported to be positively associated with muscle strength, independent of cardiorespiratory fitness (Olvera-Rojas et al., 2023). Plasma FcRL2 is mainly expressed by memory B cells and negatively regulates the B-cell receptor (BCR) pathway (Rostamzadeh et al., 2018; Tanaka and Baba, 2020; Jackson et al., 2010). Such negative regulation is important for preventing excess autoimmune reactions (Tanaka and Baba, 2020; Daëron, 2016). While the role of B-cell immunity in PD pathogenesis is not known, past studies have suggested a potential temporal relationship between decreased B-cell count and PD (Scott et al., 2023; Song et al., 2024). Magnetic resonance imaging also showed that people with higher levels of neurodegeneration, defined by brain parenchymal fraction and black hole fraction, have lower levels of peripheral FcRL2 expression (Comabella et al., 2016). Therefore, our study may provide further evidence that PA may help improve patient's brain pathology through B-cell immunity regulation.

We found a non-linear (mostly positively monotonic) relationship between strenuous MET score and NK cell proportions. PA interventions (Mathot et al., 2021; Salimans et al., 2022) have shown positive associations between repeated exercise and immune cell function. In particular, clinical trials among elderly populations suggested that moderate and intensive exercise training is positively associated with NK cell activity (Flynn et al., 1999; McFarlin et al., 2005; Oh et al., 2021). This, however, may depend on the type of physical exercise, as results are less consistent for resistance training (Sellami et al., 2018). NK cells are considered a critical modulator of inflammation for their ability to release pro-inflammatory cytokines (e.g., INF-γ) and induce pro-inflammatory M1 macrophage differentiation (Lünemann et al., 2009; Zitti and Bryceson, 2018). While the role of NK cells in PD pathology remains elusive, it has been hypothesized that they can enter the CNS following chemokine signals produced by microglia (Shi et al., 2011; Earls and Lee, 2020). Additionally, an in-vivo experimental study demonstrated that NK cells reduce α-synuclein aggregates via the endosomal/lysosomal pathway, and their depletion is associated with increased neuropathology (Earls et al., 2020). Therefore, an increased estimated proportion of NK cells may imply a neuroprotective effect against PD pathology.

Similar to the NK cell result, we found that strenuous PA is associated with a slight increase in the estimated proportion of CD8T cells. PA intervention studies among non-PD populations partially corroborate this finding, where long-term moderate-to-intense training alleviates the senescence of peripheral CD8T cells (Silva et al., 2016; Spielmann et al., 2011). In PD, emerging evidence has suggested that the infiltration of peripheral CD4T and CD8T into the CNS may play a critical role in inducing neuroinflammation. A recent study of early-to-mid-stage PD patients also revealed that, compared to healthy controls, PD patients tend to exhibit enhanced cytotoxicity and late-stage differentiation in their CD8T profiles, while the total CD8T count remained comparable (Capelle et al., 2023). Our result shows that granzyme A (GzmA), one of the most abundant extracellular cytotoxic effector molecules released by CD8T cells (Capelle et al., 2023; Garzón-Tituaña et al., 2021), did not differ by PA status, which may serve as an indication of the cytotoxicity of CD8T cells. However, further information on other effector modules (e.g., granzyme B, perforin, and granulysin) would be needed to assess whether there is an increase in CD8T cytotoxicity.

Lastly, we observed an association between moderate MET score and estimated monocyte proportion among active patients. Our finding aligns with another intervention study among PD patients, which observed a mild increase in monocytes after a 12-week exercise class program (Hu et al., 2020). Past literature has suggested that moderate (but not strenuous) exercise is linked to a reduction in the pro-inflammatory subtypes of monocytes (i.e., CD14++/CD16+ and CD14++/CD16-) and a suggestive increase in anti-inflammatory subtype (i.e., CD14+/CD16++) (Mura et al., 2022). However, we cannot determine whether such a shift in monocyte subtypes exists in our population.

Together, our study results have several implications. First, building on established literature (Conole et al.; Lu et al., 2022), our study confirms that DNAm proxies are efficient at assessing chronic inflammation when it is not feasible to obtain repeated measurements of serum markers over time. Second, our study results further confirm the beneficial influence of an active lifestyle that incorporates physical activities into daily routines (beyond planned exercise) that may be able to alleviate chronic inflammation and its harmful effects among PD patients. From a clinical perspective, in the future, it might be valuable to conduct repeated measures of these DNAm proxies in PD populations that undergo PA-focused interventions. Such studies may further elucidate the role of PA in peripheral inflammation and PD progression.

This study has some notable strengths. First, compared to intervention studies, which often enroll patients from tertiary clinics and are highly selective, we examined the associations of PA and inflammatory markers in a population-based study with real-world PA scenarios. Also, PD diagnoses and motor function assessments were made by UCLA movement disorder specialists, which differs from most epidemiologic investigations. Importantly, we constructed and used DNAm-based proxies for inflammatory markers, and it has been shown that these are more reflective of longer-term differences in cytokines compared to one-time serum protein measures (Houseman et al., 2012).

A main limitation of this study is the limited selection of available inflammation markers. While we included more than 20 DNAm cytokine proxies in our PCA, some commonly used biomarkers are absent since no methylation proxies have been developed, the most notable examples being IL-10 and TNF-α. Instead, we included DNAm cytokines proxies or immune cell estimates that are involved in the same or similar immune responses (e.g., TNF-β is structurally similar to TNF-α) (Li et al., 2021). We also included cytokines that are in the same inflammatory pathway (e.g., both IL-6 and CRP are associated with the expression of IL-10 (Sproston and Ashworth, 2018)). Another limitation of our study is that PA was self-reported, making it susceptible to reporting bias. In other words, misclassification of the activity status might occur due to faulty reporting (i.e., participants may misremember and either over- or underestimate the amount of exercise they engaged in). Given that such bias is unrelated to the measured DNA methylation we used to generate the DNAm cytokine proxies, this likely results in non-differential misclassification with a bias towards the null. Nevertheless, our study found associations between an active lifestyle and lower levels of chronic inflammation, adjusting for demographic characteristics and baseline motor function. Lastly, while our result did not indicate a statistical difference in immune cell proportion between active and sedentary PD cases, we cannot rule out more subtle changes in composition that were not captured by our methylation proxies. For example, an exercise intervention study among elderly women revealed that double-negative B cells decreased after a 6-week training (2 h/wk) while un-switched memory B cells increased (Papp et al., 2021). As double-negative B cells are often associated with the aging of the immune system and with autoimmune reactions (Sachinidis and Garyfallos, 2021), this result may indicate a beneficial shift of the B cell distribution without a considerable change in the total B cell count (Papp et al., 2021).

In summary, our study found that PA may affect chronic inflammation based on DNAm proxies among PD patients. Most notably, cases who engaged in PA had lower levels of CXCL9, CXCL10, CXCL11, CRP, and higher levels of FcRL2 compared to sedentary study participants with PD. The CRP level further decreased with an increasing amount of strenuous activity. Further, strenuous PA may also exert its beneficial effect by increasing the proportion of NK and CD8T cells. Future efforts should focus on investigating the effect of PA on immune cell differentiation, as a better understanding of subtle shifts in subtypes of CD4T, CD8T, and B cells may also improve our understanding of how PA or other factors are involved in PD pathology.

## CRediT authorship contribution statement

**Yang Cheng Hu:** Writing – review & editing, Writing – original draft, Visualization, Validation, Software, Methodology, Investigation, Formal analysis, Conceptualization. **Keren Zhang:** Writing – review & editing, Data curation. **Kimberly C. Paul:** Writing – review & editing, Supervision, Methodology. **Jin Zhou:** Writing – review & editing. **Jeff M. Bronstein:** Writing – review & editing, Data curation. **Cynthia D.J. Kusters:** Writing – review & editing, Supervision, Project administration, Methodology, Conceptualization. **Beate R. Ritz:** Writing – review & editing, Supervision, Resources, Data curation, Conceptualization.

## Declaration of competing interest

The authors declare that they have no known competing financial interests or personal relationships that could have appeared to influence the work reported in this paper.

## Data Availability

The data that has been used is confidential.

## References

[bib1] Abramson J.L., Vaccarino V. (2002). Relationship between physical activity and inflammation among apparently healthy middle-aged and older US adults. Arch. Intern. Med..

[bib2] Amara A.W., Chahine L., Seedorff N., Caspell-Garcia C.J., Coffey C., Simuni T. (2019). Self-reported physical activity levels and clinical progression in early Parkinson's disease. Parkinsonism Relat. Disorders.

[bib3] Baron U., Werner J., Schildknecht K. (2018). Epigenetic immune cell counting in human blood samples for immunodiagnostics. Sci. Transl. Med..

[bib4] Beavers K.M., Brinkley T.E., Nicklas B.J. (2010). Effect of exercise training on chronic inflammation. Clin. Chim. Acta.

[bib5] Callahan V., Hawks S., Crawford M.A. (2021). The pro-inflammatory chemokines cxcl9, cxcl10 and cxcl11 are upregulated following sars-cov-2 infection in an akt-dependent manner. Viruses.

[bib6] Camargo A. (2022). PCAtest: testing the statistical significance of Principal Component Analysis in R. PeerJ.

[bib7] Capelle C.M., Ciré S., Hedin F. (2023). Early-to-mid stage idiopathic Parkinson's disease shows enhanced cytotoxicity and differentiation in CD8 T-cells in females. Nat. Commun..

[bib8] Cebrián C., Loike J.D., Sulzer D. (2014). Neuroinflammation in Parkinson's disease animal models: a cell stress response or a step in neurodegeneration?. Curr Top Behav Neurosci.

[bib9] Chang K.P., Wu C.C., Fang K.H. (2013). Serum levels of chemokine (C-X-C motif) ligand 9 (CXCL9) are associated with tumor progression and treatment outcome in patients with oral cavity squamous cell carcinoma. Oral Oncol..

[bib10] Comabella M., Cantó E., Nurtdinov R. (2016). MRI phenotypes with high neurodegeneration are associated with peripheral blood B-cell changes. Hum. Mol. Genet..

[bib11] Conole ELS, Stevenson AJ, Green C, et al. An epigenetic proxy of chronic inflammation outperforms serum levels as a biomarker of brain ageing. medRxiv. October 2020:2020.10.08.20205245. doi:10.1101/2020.10.08.20205245.

[bib12] Contaldi E., Magistrelli L., Comi C. (2022). T lymphocytes in Parkinson's disease. J. Parkinsons Dis..

[bib13] da Silveira M.P., da Silva Fagundes K.K., Bizuti M.R., Starck É., Rossi R.C., de Resende e Silva D.T. (2021). Physical exercise as a tool to help the immune system against COVID-19: an integrative review of the current literature. Clin. Exp. Med..

[bib14] Daëron M. (2016). Fc receptors and Fc receptor-like molecules within the immunoreceptor family. Encycl Immunobiol.

[bib15] Dorans K.S., Massa J., Chitnis T., Ascherio A., Munger K.L. (2016). Physical activity and the incidence of multiple sclerosis. Neurology.

[bib16] Du Clos T.W., Mold C. (2004). C-reactive protein: an activator of innate immunity and a modulator of adaptive immunity. Immunol. Res..

[bib17] Duarte Folle A., Paul K.C., Bronstein J.M., Keener A.M., Ritz B. (2019). Clinical progression in Parkinson's disease with features of REM sleep behavior disorder: a population-based longitudinal study. Parkinsonism Relat. Disorders.

[bib18] Earls R.H., Lee J.K. (2020). The role of natural killer cells in Parkinson's disease. Exp. Mol. Med..

[bib19] Earls R.H., Menees K.B., Chung J. (2020). NK cells clear α-synuclein and the depletion of NK cells exacerbates synuclein pathology in a mouse model of α-synucleinopathy. Proc Natl Acad Sci U S A..

[bib20] Elemam N.M., Talaat I.M., Maghazachi A.A. (2022). CXCL10 chemokine: a critical player in rna and DNA viral infections. Viruses.

[bib21] Esposito K., Pontillo A., Di Palo C. (2003). Effect of weight loss and lifestyle changes on vascular inflammatory markers in obese women: a randomized trial. JAMA.

[bib22] Falck R.S., Davis J.C., Best J.R., Crockett R.A., Liu-Ambrose T. (2019). Impact of exercise training on physical and cognitive function among older adults: a systematic review and meta-analysis. Neurobiol. Aging.

[bib23] Flynn M.G., Fahlman M., Braun W.A. (1999). Effects of resistance training on selected indexes of immune function in elderly women. J. Appl. Physiol..

[bib24] Ford E.S. (2002). Does exercise reduce inflammation? Physical activity and C-reactive protein among U.S. Adults. Epidemiology (Camb., Mass.).

[bib25] Frankel H.C., Han J., Li T., Qureshi A.A. (2012). The association between physical activity and the risk of incident psoriasis. Arch. Dermatol..

[bib26] Gadd D.A., Hillary R.F., McCartney D.L. (2022). Epigenetic scores for the circulating proteome as tools for disease prediction. eLife.

[bib27] Gao H.M., Hong J.S., Zhang W., Liu B. (2002). Distinct role for microglia in rotenone-induced degeneration of dopaminergic neurons. J. Neurosci..

[bib28] Garzón-Tituaña M., Sierra-Monzón J.L., Comas L. (2021). Granzyme A inhibition reduces inflammation and increases survival during abdominal sepsis. Theranostics.

[bib29] Gelaye B., Revilla L., Lopez T. (2010). Association between insulin resistance and c-reactive protein among Peruvian adults. Diabetol. Metab. Syndr..

[bib30] Goetz C.G., Tilley B.C., Shaftman S.R. (2008). Movement disorder society-sponsored revision of the unified Parkinson's disease rating Scale (MDS-UPDRS): Scale presentation and clinimetric testing results. Mov. Disord..

[bib31] Greenland J.C., Williams-Gray C.H., Barker R.A. (2019). The clinical heterogeneity of Parkinson's disease and its therapeutic implications. Eur. J. Neurosci..

[bib32] Griffith J.W., Sokol C.L., Luster A.D. (2014). Chemokines and chemokine receptors: positioning cells for host defense and immunity. Annu. Rev. Immunol..

[bib33] Gu Y., Vorburger R., Scarmeas N. (2017). Circulating inflammatory biomarkers in relation to brain structural measurements in a non-demented elderly population. Brain Behav. Immun..

[bib34] Hald A., Lotharius J. (2005). Oxidative stress and inflammation in Parkinson's disease: is there a causal link?. Exp. Neurol..

[bib35] Hartmann A. (2004). Postmortem studies in Parkinson's disease. Dialogues Clin. Neurosci..

[bib36] Hernán M.A., Takkouche B., Caamaño-Isorna F., Gestal-Otero J.J. (2002). A meta-analysis of coffee drinking, cigarette smoking, and the risk of Parkinson's disease. Ann. Neurol..

[bib37] Horvath S. (2013). DNA methylation age of human tissues and cell types. Genome Biol..

[bib38] Horvath S. (2013). DNA methylation age of human tissues and cell types. Genome Biol..

[bib39] Houseman E.A., Accomando W.P., Koestler D.C. (2012). DNA methylation arrays as surrogate measures of cell mixture distribution. BMC Bioinf..

[bib106] Hu Y.C., Kusters C.D.J., Paul K.C. (2024). Lifetime physical activity influences Parkinson’s disease progression. Parkinsonism Relat. Disord..

[bib40] Hu Y., Zhang K., Zhang T. (2020). Exercise reverses dysregulation of T-cell-related function in blood leukocytes of patients with Parkinson's disease. Front. Neurol..

[bib41] Ishiuchi Y., Sato H., Tsujimura K. (2018). Skeletal muscle cell contraction reduces a novel myokine, chemokine (C-X-C motif) ligand 10 (CXCL10): potential roles in exercise-regulated angiogenesis. Biosci. Biotechnol. Biochem..

[bib42] Ishiuchi-Sato Y., Nedachi T. (2021). Possible involvement of CXC motif chemokine ligand 10 in exercise-induced collagen production of mouse dermal fibroblasts. Endocr. J..

[bib43] Jackson T.A., Haga C.L., Ehrhardt G.R.A., Davis R.S., Cooper M.D. (2010). FcR-like 2 inhibition of B cell receptor-mediated activation of B cells. J. Immunol..

[bib44] Kang G.A., Bronstein J.M., Masterman D.L., Redelings M., Crum J.A., Ritz B. (2005). Clinical characteristics in early Parkinson's disease in a central California population-based study. Mov. Disord..

[bib45] Kasapis C., Thompson P.D. (2005). The effects of physical activity on serum C-reactive protein and inflammatory markers: a systematic review. J. Am. Coll. Cardiol..

[bib46] Kelly A.S., Wetzsteon R.J., Kaiser D.R., Steinberger J., Bank A.J., Dengel D.R. (2004). Inflammation, insulin, and endothelial function in overweight children and adolescents: the role of exercise. J. Pediatr..

[bib47] Khalili H., Ananthakrishnan A.N., Konijeti G.G. (2013). Physical activity and risk of inflammatory bowel disease: prospective study from the Nurses' Health Study cohorts. BMJ.

[bib48] Kim S.D., Yeun Y.R. (2022). Effects of resistance training on C-reactive protein and inflammatory cytokines in elderly adults: a systematic review and meta-analysis of randomized controlled trials. Int. J. Environ. Res. Publ. Health.

[bib49] Kondo N., Nomura M., Nakaya Y., Ito S., Ohguro T. (2005). Association of inflammatory marker and highly sensitive C-reactive protein with aerobic exercise capacity, maximum oxygen uptake and insulin resistance in healthy middle-aged volunteers. Circ. J..

[bib50] Le W.D., Rowe D., Xie W., Ortiz I., He Y., Appel S.H. (2001). Microglial activation and dopaminergic cell injury: an in vitro model relevant to Parkinson's disease. J. Neurosci..

[bib51] Leal M.C., Casabona J.C., Puntel M., Pitossi F. (2013). Interleukin-1β and tumor necrosis factor-α: reliable targets for protective therapies in Parkinson's Disease?. Front. Cell. Neurosci..

[bib52] Li K., Qiu H., Yan J. (2021). The involvement of TNF-α and TNF-β as proinflammatory cytokines in lymphocyte-mediated adaptive immunity of Nile tilapia by initiating apoptosis. Dev. Comp. Immunol..

[bib53] Liu M., Guo S., Stiles J.K. (2011). The emerging role of CXCL10 in cancer. Oncol. Lett..

[bib54] Lu A.T., Binder A.M., Zhang J. (2022). DNA methylation GrimAge version 2. Aging (Albany NY).

[bib55] Lünemann A., Lünemann J.D., Münz C. (2009). Regulatory NK-cell functions in inflammation and autoimmunity. Mol Med.

[bib56] Luo B., Xiang D., Ji X. (2024). The anti-inflammatory effects of exercise on autoimmune diseases: a 20-year systematic review. J Sport Heal Sci..

[bib57] Marcos-Pérez D., Cruces-Salguero S., García-Domínguez E. (2024). Physical interventions restore physical frailty and the expression of CXCL-10 and IL-1β inflammatory biomarkers in old individuals and mice. Biomolecules.

[bib58] Marsland A.L., Gianaros P.J., Kuan D.C.H., Sheu L.K., Krajina K., Manuck S.B. (2015). Brain morphology links systemic inflammation to cognitive function in midlife adults. Brain Behav. Immun..

[bib59] Mathot E., Liberman K., Cao Dinh H., Njemini R., Bautmans I. (2021). Systematic review on the effects of physical exercise on cellular immunosenescence-related markers – an update. Exp. Gerontol..

[bib60] Mayer-Davis E.J., D'Agostino R., Karter A.J. (1998). Intensity and amount of physical activity in relation to insulin sensitivity: the Insulin Resistance Atherosclerosis Study. JAMA.

[bib61] McFarlin B.K., Flynn M.G., Phillips M.D., Stewart L.K., Timmerman K.L. (2005). Chronic resistance exercise training improves natural killer cell activity in older women. Journals Gerontol Ser A.

[bib62] McGeer P.L., McGeer E.G. (2004). Inflammation and neurodegeneration in Parkinson's disease. Parkinsonism Relat. Disorders.

[bib63] Metsios G.S., Moe R.H., Kitas G.D. (2020). Exercise and inflammation. Best Pract. Res. Clin. Rheumatol..

[bib64] Mura M., Weiss-Gayet M., Della-Schiava N. (2022). Monocyte phenotypes and physical activity in patients with carotid atherosclerosis. Antioxidants.

[bib65] Nehring S.M., Goyal A., Bansal P., Patel B.C. (2023). C reactive protein. StatPearls.

[bib66] Oh S., Chun S., Hwang S. (2021). Vitamin D and exercise are major determinants of natural killer cell activity, which is age- and gender-specific. Front. Immunol..

[bib67] Olvera-Rojas M., Plaza-Florido A., Solis-Urra P. (2023). Association of muscular strength and targeted proteomics involved in brain health in children with overweight/obesity. Scand. J. Med. Sci. Sports.

[bib68] Papp G., Szabó K., Jámbor I. (2021). Regular exercise may restore certain age-related alterations of adaptive immunity and rebalance immune regulation. Front. Immunol..

[bib69] Paul K.C., Rausch R., Creek M.M. (2016). APOE, MAPT, and COMT and Parkinson's disease susceptibility and cognitive symptom progression. J. Parkinsons Dis..

[bib70] Paul K.C., Chuang Y.H., Shih I.F. (2019). The association between lifestyle factors and Parkinson's disease progression and mortality. Mov. Disord..

[bib71] Poewe W., Seppi K., Tanner C.M. (2017). Parkinson disease. Nat. Rev. Dis. Primers.

[bib72] Ritz B.R., Paul K.C., Bronstein J.M. (2016). Of pesticides and men: a California story of genes and environment in Parkinson's disease. Curr Environ Heal reports.

[bib73] Romeo J., Wärnberg J., Pozo T., Marcos A. (2010). Physical activity, immunity and infection. Proc. Nutr. Soc..

[bib74] Rose G.L., Skinner T.L., Mielke G.I., Schaumberg M.A. (2021). The effect of exercise intensity on chronic inflammation: a systematic review and meta-analysis. J. Sci. Med. Sport.

[bib75] Rostamzadeh D., Kazemi T., Amirghofran Z., Shabani M. (2018). Update on Fc receptor-like (FCRL) family: new immunoregulatory players in health and diseases. Expert Opin. Ther. Targets.

[bib76] Ruuskanen O., Luoto R., Valtonen M., Heinonen O.J., Waris M. (2022). Respiratory viral infections in athletes: many unanswered questions. Sport Med.

[bib77] Sachinidis A., Garyfallos A. (2021). Double Negative (DN) B cells: a connecting bridge between rheumatic diseases and COVID-19?. Mediterr J Rheumatol.

[bib78] Salimans L., Liberman K., Njemini R., Kortekaas Krohn I., Gutermuth J., Bautmans I. (2022). The effect of resistance exercise on the immune cell function in humans: a systematic review. Exp. Gerontol..

[bib79] Scott K.M., Chong Y.T., Park S. (2023). B lymphocyte responses in Parkinson's disease and their possible significance in disease progression. Brain Commun.

[bib80] Sellami M., Gasmi M., Denham J. (2018). Effects of acute and chronic exercise on immunological parameters in the elderly aged: can physical activity counteract the effects of aging?. Front. Immunol..

[bib81] Shi F.D., Ljunggren H.G., La Cava A., Van Kaer L. (2011). Organ-specific features of natural killer cells. Nat. Rev. Immunol..

[bib82] Shih I.F., Liew Z., Krause N., Ritz B. (2016). Lifetime occupational and leisure time physical activity and risk of Parkinson's disease. Parkinsonism Relat. Disorders.

[bib83] Shih I.F., Paul K., Haan M., Yu Y., Ritz B. (2018). Physical activity modifies the influence of APOE ε4 allele and type 2 diabetes on dementia and cognitive impairment among older Mexican Americans. Alzheimer's Dement..

[bib84] Sieczkowska S.M., Smaira F.I., Mazzolani B.C., Gualano B., Roschel H., Peçanha T. (2021). Efficacy of home-based physical activity interventions in patients with autoimmune rheumatic diseases: a systematic review and meta-analysis. Semin. Arthritis Rheum..

[bib85] Silva L.C.R., de Araújo A.L., Fernandes J.R. (2016). Moderate and intense exercise lifestyles attenuate the effects of aging on telomere length and the survival and composition of T cell subpopulations. Age.

[bib86] Song J., Qin Y., Wang L. (2024). Exploring the causal relationship between B lymphocytes and Parkinson's disease: a bidirectional, two-sample Mendelian randomization study. Sci Reports.

[bib87] Souza P.S., Gonçalves E.D., Pedroso G.S. (2017). Physical exercise attenuates experimental autoimmune encephalomyelitis by inhibiting peripheral immune response and blood-brain barrier disruption. Mol. Neurobiol..

[bib88] Spielmann G., McFarlin B.K., O'Connor D.P., Smith P.J.W., Pircher H., Simpson R.J. (2011). Aerobic fitness is associated with lower proportions of senescent blood T-cells in man. Brain Behav. Immun..

[bib89] Sproston N.R., Ashworth J.J. (2018). Role of C-reactive protein at sites of inflammation and infection. Front. Immunol..

[bib90] Stevenson A.J., Gadd D.A., Hillary R.F. (2021). Creating and validating a DNA methylation-based proxy for interleukin-6. Journals Gerontol Ser A Biol Sci Med Sci.

[bib91] Szymura J., Kubica J., Wiecek M., Pera J. (2020). The immunomodulary effects of systematic exercise in older adults and people with Parkinson's disease. J. Clin. Med..

[bib92] Taaffe D.R., Harris T.B., Ferrucci L., Rowe J., Seeman T.E. (2000). Cross-sectional and prospective relationships of interleukin-6 and C-reactive protein with physical performance in elderly persons: MacArthur studies of successful aging. Journals Gerontol Ser A..

[bib93] Tanaka S., Baba Y. (2020). B cell receptor signaling. Adv. Exp. Med. Biol..

[bib94] Teschendorff A.E., Marabita F., Lechner M. (2013). A beta-mixture quantile normalization method for correcting probe design bias in Illumina Infinium 450 k DNA methylation data. Bioinformatics.

[bib95] Thacker E.L., Chen H., Patel A.V. (2008). Recreational physical activity and risk of Parkinson's disease. Mov. Disord..

[bib96] Tsukita K., Sakamaki-Tsukita H., Takahashi R. (2022). Long-term effect of regular physical activity and exercise habits in patients with early Parkinson disease. Neurology.

[bib97] Vieira V.M.N.C.S. (2012). Permutation tests to estimate significances on principal components analysis. Comput Ecol Softw.

[bib98] Wang A., Cockburn M., Ly T.T., Bronstein J.M., Ritz B. (2014). The association between ambient exposure to organophosphates and Parkinson's disease risk. Occup. Environ. Med..

[bib99] Wang Q., Liu Y., Zhou J. (2015). Neuroinflammation in Parkinson's disease and its potential as therapeutic target. Transl. Neurodegener..

[bib100] Wang X., Zhang Y., Wang S. (2022). The role of CXCR3 and its ligands in cancer. Front. Oncol..

[bib101] Williams G.P., Schonhoff A.M., Sette A., Lindestam Arlehamn C.S. (2022). Central and peripheral inflammation: connecting the immune responses of Parkinson's disease. J. Parkinsons Dis..

[bib102] Xu Q., Park Y., Huang X. (2010). Physical activities and future risk of Parkinson disease. Neurology.

[bib103] Yang F., Lagerros Y.T., Bellocco R. (2015). Physical activity and risk of Parkinson's disease in the Swedish national march cohort. Brain.

[bib104] Zitti B., Bryceson Y.T. (2018). Natural killer cells in inflammation and autoimmunity. Cytokine Growth Factor Rev..

[bib105] Zoladz J.A., Majerczak J. (2014). Moderate-intensity interval training increases serum brain-derived neurotrophic factor level and decreases inflammation in Parkinson's disease patients Kisiel-Sajewicz View project kisiel-sajewicz View project. Artic J Physiol Pharmacol an Off J Polish Physiol Soc..

